# Gene-environment interactions and preterm birth predictors: A Bayesian network approach

**DOI:** 10.1590/1678-4685-GMB-2023-0090

**Published:** 2024-01-19

**Authors:** Dario E. Elias, Maria R. Santos, Hebe Campaña, Fernando A. Poletta, Silvina L. Heisecke, Juan A. Gili, Julia Ratowiecki, Viviana R. Cosentino, Rocio Uranga, Diana Rojas Málaga, Alice Brinckmann Oliveira, Ana Carolina Brusius-Facchin, César Saleme, Mónica Rittler, Hugo B. Krupitzki, Jorge S. Lopez Camelo, Lucas G. Gimenez

**Affiliations:** 1 Estudio Colaborativo Latino Americano de Malformaciones Congénitas (ECLAMC), Centro de Educación Médica e Investigaciones Clínicas-Consejo Nacional de Investigaciones Científicas y Técnicas (CEMIC-CONICET), Ciudad Autónoma de Buenos Aires, Argentina.; 2 Comisión de Investigaciones Científicas, Buenos Aires, Argentina.; 3 Instituto Multidisciplinario de Biología Celular, Buenos Aires, Argentina.; 4 Instituto Nacional de Genética Médica Populacional (INAGEMP), CEMIC-CONICET, Ciudad Autónoma de Buenos Aires, Argentina.; 5 Dirección de Investigación, CEMIC-CONICET, Ciudad Autónoma de Buenos Aires, Argentina.; 6 Instituto Académico Pedagógico de Ciencias Humanas, Universidad Nacional de Villa María, Córdoba, Argentina.; 7 Hospital Interzonal General de Agudos Luisa C. de Gandulfo, Buenos Aires, Argentina.; 8 Hospital San Juan de Dios, Buenos Aires, Argentina.; 9 Serviço de Genética Médica, Hospital de Clínicas de Porto Alegre (HCPA), Porto Alegre, Rio Grande do Sul, Brasil.; 10 Instituto de Maternidad y Ginecología Nuestra Señora de las Mercedes, Tucumán, Argentina.; 11 Hospital Materno Infantil Ramón Sardá, Ciudad Autónoma de Buenos Aires, Argentina.; 12 Instituto Universitario, Centro de Educación Médica e Investigaciones Clínicas (CEMIC-IUC), Ciudad Autónoma de Buenos Aires, Argentina.

**Keywords:** Preterm birth, gene-environment interaction, neighbourhood characteristics, toxoplasmosis, Bayesian approach

## Abstract

Preterm birth (PTB) is the main condition related to perinatal morbimortality worldwide. The aim of this study was to identify gene-environment interactions associated with spontaneous PTB or its predictors. We carried out a retrospective case-control study including parental sociodemographic and obstetric data as well as newborn genetic variants of 69 preterm and 61 at term newborns born at a maternity hospital from Tucumán, Argentina, between 2005 and 2010. A data-driven Bayesian network including the main PTB predictors was created where we identified gene-environment interactions. We used logistic regressions to calculate the odds ratios and confidence intervals of the interactions. From the main PTB predictors (nine exposures and six genetic variants) we identified an interaction between low neighbourhood socioeconomic status and rs2074351 (*PON1*, genotype GG) variant that was associated with an increased risk of toxoplasmosis (odds ratio 12.51, confidence interval 95%: 1.71 - 91.36). The results of this exploratory study suggest that structural social disparities could influence the PTB risk by increasing the frequency of exposures that potentiate the risk associated with individual characteristics such as genetic traits. Future studies with larger sample sizes are necessary to confirm these findings.

## Introduction

Preterm birth (PTB) is defined as the birth of a conceptus before 37 weeks of gestational age. The estimated global PTB rate was 10.6% in 2014, while in Argentina it was 8.7% in 2020 (Chawanpaiboon et al., 2019; Dirección de Estadísticas e Información de Salud, 2022). In 2018, 35% of worldwide neonatal deaths were associated with PTB (UNICEF *et al.*, 2019). PTB is considered a multifactorial aetiology condition where several factors such as sociodemographic aspects, habits, obstetric history, genetic traits, and health conditions are involved (Cobo et al., 2020). 

Bayesian networks (BN) are graphical probabilistic models where the nodes represent variables and the edges the conditional dependencies among them (Koller and Friedman, 2009). BN have contributed to epidemiology by facilitating visualisation and interpretation of dependencies among variables. Several methods have been developed for data-driven BN constructions; for example, score-based algorithms which explore the space of possible networks using a heuristic searching algorithm and select the BN with the best goodness of fit; constraint-based algorithms, which use conditional independence tests to learn the dependency structure of data; and hybrid algorithms that combine both approaches (Scutari et al., 2019). In addition, previous studies have proposed the use of BN for identifying gene environment (GxE) interactions (Su et al., 2013). However, to our knowledge, no GxE interaction studies have been performed for PTB using BN.

In previous works, we analysed sociodemographic, clinical, and genetic factors predisposing to PTB in an Argentine population sample (Krupitzki et al., 2013; Gimenez et al., 2016; Gimenez *et al*., 2017; Elias et al., 2021). We also used BN to analyse the association of sociodemographic and obstetric characteristics with PTB (Elias *et al*., 2022a). In another study, we carried out newborn DNA candidate genes sequencing and identified characteristics with the highest PTB predictive power; they included maternal sociodemographic and biological characteristics, neighbourhood socioeconomic status (NSES), and newborn genetic variants in *KCNN3*, *COL4A3*, *PON1*, and *CRHR1* genes (Elias *et al*., 2022b). In the present study, we created a BN with exposures and genetic variants previously related with PTB to identify GxE interactions associated with PTB or its predictors.

## Subjects and Methods

### Study design

A retrospective unmatched case-control study was conducted including women who gave birth at the Instituto de Maternidad y Ginecología Nuestra Señora de Las Mercedes, a public maternity hospital from Tucumán, Argentina. Recruitment was carried out between July 2005 and December 2010. Women eligible for the study were invited to participate after delivery and before hospital discharge. The case group comprised preterm infants born to multigravid women. The control group included infants born at term to multigravid women without a previous history of PTB nor pregnancy loss. Exclusion criteria were medically induced PTB, neonates with congenital anomalies, multiple gestation, and maternal age under 16 years. This study is part of an international collaborative project aimed at elucidating factors associated with PTB (Krupitzki et al., 2013; Gimenez et al., 2016; Gimenez *et al*., 2017; Elias et al., 2021; Elias *et al*., 2022a; Elias *et al*., 2022b).

### Data collection

Women who agreed to participate in the study were interviewed by qualified members of the Estudio Colaborativo Latino Americano de Malformaciones Congénitas (ECLAMC) (Castilla and Orioli, 2004). Data from clinical records and a structured questionnaire designed to collect information on sociodemographic aspects, maternal reproductive history, obstetric complications, and neonatal outcomes were registered in standardised research forms. All collected data were reviewed by paediatricians and obstetricians involved in the study. 

### Ethics approval

Study protocols were approved by the Centro de Educación Médica e Investigaciones Clínicas (CEMIC) Ethics Committee (IRB 00001745-IORG 0001315) and the University of Iowa Institutional Review Board (IRB 200411759). Parents provided written informed consent for themselves and the neonates.

### Outcome and exposure variables

The primary outcome variable was PTB, defined as a live birth of less than 37 gestational weeks (preterm birth: 1, at term birth: 0). The gestational age was estimated from the last menstrual period date; if uncertain, an ultrasound examination was performed before 22 weeks of estimated gestation (Dietz et al., 2007). If the difference between both methods was greater than 7 days, gestational age by ultrasound was used. Surveyed individual and contextual exposure variables and imputation method for missing data are described in Appendix S1. Appendix S2 describes the sequencing methodology and variant calling of newborns’ candidate genes. Only the exposures and newborns’ genetic variants that presented the highest PTB predictive power found in a previous study (Elias et al., 2022b ), which was conducted with the same data of the present study, were included. The exposures were maternal individual characteristics [few prenatal visits (<5), sexual activity during the last month of pregnancy, maternal blood ABO group A, gestation number, toxoplasmosis [determined from the IgG serological test performed during routine screening (Dirección Nacional de Maternidad e Infancia, 2010)], body mass index (BMI) at the beginning of pregnancy (calculated from height and self-reported weight at beginning of pregnancy), maternal age and anemia], residential context characteristics [NSES estimated on the proportion of neighborhood households without Unsatisfied Basic Needs (UBN), described in Appendix S1], and newborn genetic variants [rs4845397 (*KCNN3*), rs11680670 (*COL4A3*), rs12621551 (*COL4A3*), rs73993878 (*COL4A3*), rs2074351 (*PON1*), rs8073146 (*CRHR1*)] (Elias *et al*., 2022b). Variables that could have a moderating or confounding effect on the analyzed interactions were included in a sensitivity analysis (maternal schooling, self-reported ancestry, urinary tract infections, vaginal discharge, tobacco smoking, newborn sex, living in large urban conglomerate, and address accuracy). Continuous and ordinal variables (maternal age, gestation number, BMI, and NSES) were stratified using the 25th and 75th percentiles. Newborn genetic variants were binarized considering the presence (1) or absence (0) of at least one copy of the less frequent allele.

### Bayesian network

We created a data-driven BN based on PTB and exposures which showed the highest PTB predictive power. The BN structure was determined by a score based method which assigned a score to each candidate BN reflecting its goodness of fit and then tried to maximise it with a heuristic search algorithm (Scutari et al., 2019). We applied the tabu search algorithm that starts from an iterative greedy search process in which modifications are made to the BN (*e. g.*, remove or add an edge) and the BN score is calculated. The tabu search maintains a list of the 10 last built BN and continues searching for a better BN that has not yet been considered. Possible edge directions that were not relevant to the present study were excluded (*e. g.*, from “Preterm birth” to “Few prenatal visits”) (Table S1). Bayesian Dirichlet equivalent was used to determine the BN goodness of fit (Heckerman et al., 1995). We generated 10,000 BN by using a bootstrap method and then selected the edges that were present in at least 15% of the BN. The OR of each relationship was calculated from the conditional probabilities determined with the logic sampling method (Henrion, 1988). R packages bnlearn and igraph were used (Scutari, 2010; Csardi and Nepusz, 2006).

### Interactions

Based on the observation of the BN, interaction analyses were performed using Firth’s penalised logistic regression (Firth, 1993). Considering that it would be more likely to observe a statistical interaction between variables when their independence is greater (Su et al., 2013), the interactions to be evaluated were selected with the following criteria: given an outcome O and exposures A and B with conditional dependencies towards O in the BN, the interaction between A and B with O as outcome was analysed if there was no conditional dependence in the BN between A and B. In particular, we focused on GxE interactions of newborn genetic variants. For exposures with multiple categories (maternal age, gestation number, BMI, and NSES), we included the interaction of genetic variants with all non-reference exposure categories in the model. Based on the inspection of the BN, in the regressions we used the genotype of the genetic variants whose effect on the result would have the same direction as the exposures (*i. e.*, both increase the probability of the outcome or both decrease it). We analysed the sensitivity of the selected interactions including one covariate at a time to maintain the relationship between the number of events by the number of variables included in the models greater than 5 (Vittinghoff and McCulloch, 2007). The covariates were considered including their main effects and their interactions with the exposures and analysed genetic variants (Keller, 2014). R package logistf was used (Heinze *et al*., 2020).

## Results

In this study, data from 130 newborns (61 term and 69 preterm newborns) were analysed. Table 1 shows the frequency of the included variables.

**Table 1 -:** Frequency of variables in cases and controls. The variables that had the highest predictive power of PTB and variables included for the sensitivity analysis are shown. The variables maternal age, gestation number, BMI, and NSES were categorised using the 25th and 75th percentiles. In newborn genetic variants, the gene, region of the variant and genotypes of the less frequency allele are shown in parenthesis. Abbreviations: n, total number of newborns in the group; N, number of newborns in the category of each variable; BMI, body mass index; NSES, neighbourhood socioeconomic status; UBN, unsatisfied basic needs; UTR, untranslated region.

Variable		Total (n=130) N (%)	Case (n=69) N (%)	Control (n=61) N (%)	Chi-Squared P Value	Imputed data (%)
Maternal schooling	Low (< 7 years)	25 (19.23)	12 (17.39)	13 (21.31)	0.7316	0.00
	Middle-High (≥ 7 years)	105 (80.77)	57 (82.61)	48 (78.69)	0.7316	
Maternal age	Low (< 24 years)	37 (28.46)	25 (36.23)	12 (19.67)	0.0583	0.00
	Middle (24 - 31 years)	95 (73.08)	56 (81.16)	39 (63.93)	0.0443	
	High (> 31 years)	35 (26.92)	13 (18.84)	22 (36.07)	0.0443	
BMI at the beginning of pregnancy	Low (< 21.0 kg/m^2^)	33 (25.38)	25 (36.23)	8 (13.11)	0.0048	4.62
	Middle (21.0 - 26.6 kg/m^2^)	64 (49.23)	30 (43.48)	34 (55.74)	0.2226	
	High (>26.6 kg/m^2^)	33 (25.38)	14 (20.29)	19 (31.15)	0.2234	
Number of gestation	Low (< 4)	50 (38.46)	29 (42.03)	21 (34.43)	0.4786	0.00
	Middle (4 - 5)	46 (35.38)	27 (39.13)	19 (31.15)	0.4436	
	High (> 5)	34 (26.15)	13 (18.84)	21 (34.43)	0.0691	
Non-native ancestry		16 (12.31)	9 (13.04)	7 (11.48)	0.9967	0.00
Maternal blood ABO group A		41 (31.54)	28 (40.58)	13 (21.31)	0.0300	2.31
Sexual activity during the last month of pregnancy		36 (27.69)	28 (40.58)	8 (13.11)	0.0010	0.77
Few prenatal visits (<5)		62 (47.69)	41 (59.42)	21 (34.43)	0.0076	1.54
Toxoplasmosis		44 (33.85)	30 (43.48)	14 (22.95)	0.0224	16.92
Urinary tract infection		42 (32.31)	22 (31.88)	20 (32.79)	1.0000	2.31
Vaginal discharge		44 (33.85)	27 (39.13)	17 (27.87)	0.2426	0.00
Anaemia		58 (44.62)	36 (52.17)	22 (36.07)	0.0955	16.92
Tobacco smoking	before pregnancy	53 (40.77)	26 (37.68)	27 (44.26)	0.5597	0.00
	during pregnancy	34 (26.15)	18 (26.09)	16 (26.23)	1.0000	0.00
Passive smoking	52 (40.00)	24 (34.78)	28 (45.90)	0.2661	0.00	
Alcohol intake	before pregnancy	30 (23.08)	19 (27.54)	11 (18.03)	0.2824	0.00
	during pregnancy	12 (9.23)	9 (13.04)	3 (4.92)	0.1958	3.85
Male newborn sex		61 (46.92)	30 (43.48)	31 (50.82)	0.5086	0.00
rs4845397 (*KCNN3*, 5’ UTR, GT: TC or TT)		36 (27.69)	16 (23.19)	20 (32.79)	0.3058	2.31
rs12621551 (*COL4A3*, intron, GT: TG or TT)		48 (36.92)	23 (33.33)	25 (40.98)	0.4716	0.77
rs73993878 (*COL4A3*, intron, GT: AG or AA)		22 (16.92)	13 (18.84)	9 (14.75)	0.6997	0.00
rs11680670 (*COL4A3*, intron, GT: TC or TT)		51 (39.23)	26 (37.68)	25 (40.98)	0.8377	0.77
rs2074351 (*PON1*, intron, GT: AG or AA)		59 (45.38)	30 (43.48)	29 (47.54)	0.7735	0.77
rs8073146 (*CRHR1*, intron, GT: GA or GG)		24 (18.46)	9 (13.04)	15 (24.59)	0.1424	0.77
NSES (% of neighbourhood households without UBN)	Low (< 79.2%)	33 (25.38)	19 (27.54)	14 (22.95)	0.6909	0.00
	Middle (79.2% - 88.0%)	64 (49.23)	37 (53.62)	27 (44.26)	0.3737	
	High (> 88.0%)	33 (25.38)	13 (18.84)	20 (32.79)	0.1049	
Lives in the largest urban conglomerate		79 (60.77)	41 (59.42)	38 (62.30)	0.8768	0.00
Domicile accuracy at the neighbourhood level		66 (50.77)	35 (50.72)	31 (50.82)	1.0000	0.00

**Figure 1 -:**
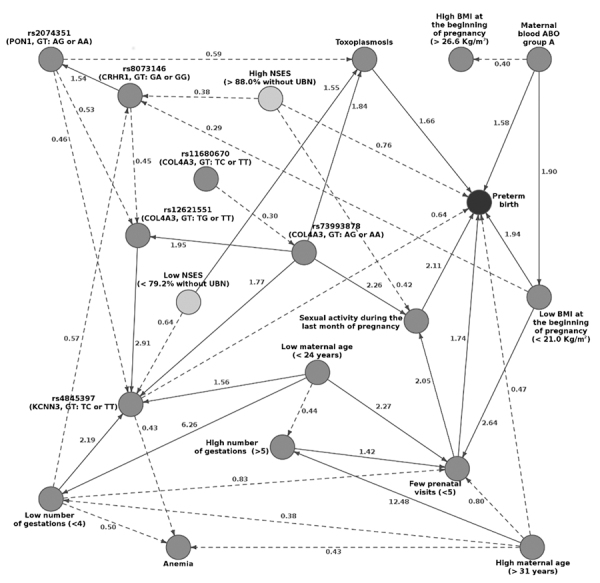
Bayesian network of preterm birth predictors. The nodes represent the variables and the edges the conditional dependencies between them. In dark grey the preterm birth variable and, in light grey, NSES variables. The edge numbers are the estimated odds ratios; dashed and solid edges correspond to odds ratios less and greater than 1, respectively. Abbreviations: BMI: body mass index; GT: genotype; NSES: neighbourhood socioeconomic status; PTB: preterm birth; UBN: unsatisfied basic needs.

The BN created with the selected predictors presented 20 nodes and 42 edges (Figure 1). Table 2 shows the possible interactions perceived through the BN inspection. Only the interaction between low NSES and rs2074351 (*PON1*, genotype: GG) variant with toxoplasmosis as outcome presented an OR different from 1 with a 95% CI (Table 2 and 3). The interaction between low NSES and the rs2074351 variant was greater than one with a 95% CI considering as covariates the rest of the exposures and genetic variants listed in Table 1 (Table S2). The frequency of toxoplasmosis by NSES category was 42.4% (14/33), 34.4% (22/64), and 24.2% (8/33) for low, medium, and high categories, respectively; the Chi-square P Value was 0.29.

**Table 2 -:** Gene-environment interactions evaluated from the inspection of the BN. Only the odds ratio of the interaction term is shown. Abbreviations: BN, Bayesian network; CI, confidence interval; FDR, false discovery rate; GT: genotype; NSES, neighbourhood socioeconomic status.

Outcome	Interaction term	Odds ratio (95% CI)	P Value	FDR
Preterm birth	Few prenatal visits : rs4845397 (*KCNN3*, GT: CC)	1.65 (0.32, 8.34)	0.5472	0.7296
	Maternal blood ABO group A : rs4845397 (*KCNN3*, GT: CC)	2.38 (0.44, 12.99)	0.3169	0.5070
	Low BMI at the beginning of pregnancy : rs4845397 (*KCNN3*, GT: CC)	1.51 (0.20, 11.53)	0.6913	0.7900
	Sexual activity during the last month of pregnancy : rs4845397 (*KCNN3*, GT: CC)	0.24 (0.03, 1.73)	0.1567	0.4179
	Toxoplasmosis : rs4845397 (*KCNN3*, GT: CC)	0.23 (0.03, 1.53)	0.1276	0.4179
Toxoplasmosis	Low NSES : rs73993878 (*COL4A3*, GT: AG or AA)	4.59 (0.36, 58.81)	0.2415	0.4829
	Low NSES : rs2074351 (*PON1*, GT: GG)	12.51 (1.71, 91.36)	0.0127	0.1018
Sexual activity during the last month of pregnancy	High NSES : rs73993878 (*COL4A3*, GT: GG)	0.83 (0.07, 9.33)	0.8785	0.8785

**Table 3 -:** Odds ratios of the interaction between NSES and rs2074351 (*PON1*, GT: GG) for toxoplasmosis. Abbreviations: CI, confidence interval; GT: genotype; NSES, neighbourhood socioeconomic status.

Term	OR (95% CI)	P Value	Toxoplasmosis	
			No (n=86) N (%)	Yes (n=44) N (%)
Low NSES	0.31 (0.06, 1.52)	0.1503	13 (15.1)	2 (4.5)
rs2074351 (*PON1*, GT: GG)	0.83 (0.30, 2.32)	0.7225	23 (26.7)	11 (25.0)
High NSES	0.52 (0.12, 2.18)	0.3690	11 (12.8)	3 (6.8)
Low NSES : rs2074351 (*PON1*, GT: GG)	12.51 (1.71, 91.36)	0.0127	6 (7.0)	12 (27.3)
High NSES : rs2074351 (*PON1*, GT: GG)	1.50 (0.22, 10.04)	0.6747	14 (16.3)	5 (11.4)
Reference [middle NSES and rs2074351 (*PON1*, GT: AG or AA)]	-	-	19 (22.1)	11 (25.0)

## Discussion

From the construction of a BN with PTB predictors, an interaction between rs2074351 (*PON1*) and low NSES was identified, which was associated with an increased risk of toxoplasmosis.

Toxoplasmosis is an infection caused by an intracellular parasite called *Toxoplasma gondii* (*T. gondii*). It is one of the most prevalent infections and is estimated to affect a third of the world population (Ahmed et al., 2020). Infection during pregnancy can affect the foetus resulting in congenital toxoplasmosis that is associated with PTB and conditions such as newborn´s neurological disease and blindness (Ahmed *et al*., 2020). Transmission of *T. gondii* to humans generally occurs through ingestion of tissue cysts contained in contaminated undercooked meat products. It can also be transmitted by consumption of water or vegetables contaminated with infected cat or mice faeces. *T. gondii* infections are largely asymptomatic during the acute and chronic phases, with the chronic phase persisting during the host’s whole life. *T. gondii* tachyzoites and bradyzoites replicate intracellularly and acquire nutrients from their host cells such as lipids and their precursors (Blume and Seeber, 2018; Ahmed *et al*., 2020).

Regarding the prevalence of toxoplasmosis in pregnant women in Argentina, Carral et al. (2008) reported a prevalence of specific IgG anti-*T. gondii* antibodies of 49% in pregnant women treated in maternity hospitals of Ciudad Autónoma de Buenos Aires and of Provincia de Buenos Aires. More recently, a prevalence of 18.33% was reported in a hospital of the Ciudad Autónoma de Buenos Aires (Carral *et al*., 2013). In addition, a higher prevalence was reported in peri-urban areas (36.4%) than in urban areas (26.8%) of Provincia de Buenos Aires (Rivera et al., 2019).

Mareze et al. (2019) reported higher risk of toxoplasmosis in populations with low socioeconomic level while Ncube et al. (2016) informed higher frequencies of adverse birth outcomes in low NSES. Women living in low NSES have less access to healthy food, health services, leisure activities, and social support, while their exposure to poor air and water quality, and to societal stressors is greater (Diez Roux and Mair, 2010). In this work, we used the UBN index to define the NSES categories. The UBN is a poverty direct measurement method that relates well-being to actual consumption. The UBN index defines minimum welfare thresholds and has been used in Latin American studies since the 1980s (Feres and Mancero, 2001). One of the strengths of this index is that it can be calculated from census data, allowing it to take advantage of the geographic disaggregation provided by the census information. However, the UBN index also has some limitations; for example, although it allows distinguishing households with and without critical deficiencies, it does not allow to identify their magnitude. It neither allows the identification of recent poverty situations nor to measure current income or expenses, which are usually analysed with other methods such as the poverty line. In addition, the UBN index has a certain sensitivity to differentiate urban and rural populations (Feres and Mancero, 2001).

Human serum paraoxonase-1 (PON1) is a calcium-dependent hydrolytic enzyme. Paraoxonases are a component of the immune system and their response to infections is related to the inhibition of plasma lipid oxidation and decreasing levels of proteins involved in the HDL-mediated cholesterol reverse transport (Camps et al., 2017). Previous studies have shown a lower expression of *PON1* in pregnant women with chorioamnionitis (Soydinç et al., 2012). PON1 also has detoxification functions; it acts as an A-esterase capable of hydrolyzing the active metabolites (oxons) of various organophosphate pesticides (Costa et al., 2013). Several studies have identified modulators of PON1 activity and *PON1* expression such as exposure to carbon monoxide, arsenic, lead, and tobacco smoke (Costa *et al*., 2005; Li et al., 2006; Li *et al*., 2009; Haj Mouhamed et al., 2012; Zengin et al., 2014). Likewise, certain genetic variants in *PON1* are also involved in its expression and in the PON1 activity. For example, the Q192R polymorphism is associated with a differential catalytic activity on some organophosphate substrates while the polymorphism at position -108 (C/T) is the main contributor to the differences in *PON1* expression levels (Costa *et al*., 2013).

In this study, an interaction between low NSES and the rs2074351 (*PON1*) variant, associated with a higher risk of toxoplasmosis was observed. The rs2074351 variant, present in an intronic region, could affect the expression of *PON1* possibly decreasing the immune system response (Jo and Choi, 2015), which increases susceptibility to infections. Such susceptibility would be higher in areas of low NSES where a higher frequency of *T. gondii* in the environment can be expected, as well as that of other exposures that affect PON1 activity or *PON1* expression. In this way, it could be understood that the interaction between the *PON1* rs2074351 variant and the low NSES context presented a higher risk of toxoplasmosis than their individual effects. It also suggests that structural social disparities, in addition to their direct and indirect effects on PTB risk (*e. g.* access to healthcare services) (Elias et al., 2022 a ), might influence PTB risk by increasing the frequency of exposures that potentiate the risk associated with individual characteristics, such as genetic traits.

Further studies are required to analyse maternal genotypes and to identify other exposures linked to NSES and the extent to which they may affect PON1 activity or *PON1* expression. For example, considering the modulators of PON1 activity, air pollutants produced by the burning of cane fields and the presence of water pollutants such as pesticides and arsenic have been reported to exist near the study population (Guber et al., 2009; Piriz Carrillo et al., 2010; Chaile et al., 2011).

The reader of this article should bear in mind the following limitations. Although the small sample size allowed the exploratory nature of this work, further studies with larger sample size are necessary (Vittinghoff and McCulloch, 2007). The categorization of certain variables (*e. g.* maternal age) was based on the 25th and 75th percentiles of their distribution because the sample size did not allow the use of usual categories (such as maternal age <20 years); this aspect may limit the comparison with other studies. Women were recruited from a single maternity hospital; multicenter studies including more heterogeneous populations may reveal other interactions. Although the diagnosis of toxoplasmosis was based on a serological test, the time of exposure could not be defined. Finally, 2.02% of the data of this study were imputed.

In conclusion, based on the used methodology, the results of this study showed that the interaction between a *PON1* variant and low NSES was associated with an increased risk of toxoplasmosis, suggesting that contextual and individual characteristics interact to increase the risk of infections which, in turn, can increase the chances of PTB. Structural social disparities could influence the PTB risk by increasing the frequency of exposures that potentiate the risk associated with individual characteristics such as genetic traits. Future studies with larger sample sizes are necessary to confirm these findings and to analyse a greater number of exposures.

## Supplementary material

The following online material is available for this article:

Appendix S1 - Individual and contextual data.

Appendix S2 - Sequencing and variant calling of newborn genes.

Table S1 - Potential edge directions excluded from Bayesian network structure learning.

Table S2 - Regression models tested with the interaction between rs2074351 (*PON1*) and NSES for toxoplasmosis.
