# Supplementary material for: Gene-environment interactions and preterm birth predictors: A Bayesian network approach
Source: Genet Mol Biol. 2024 Jan 19;46(4):e20230090. doi: 10.1590/1678-4685-GMB-2023-0090 (PMC10804443; doi:10.1590/1678-4685-GMB-2023-0090)
Supplement: Appendix S1 - [file 1415-4757-GMB-46-4-e20230090-s1.pdf]

Genetics and Molecular Biology
https://doi.org/10.1590/1678-4685-GMB-2023-0090

Supplementary Material to “Gene-environment interactions and preterm birth predictors: A Bayesian network approach”

Appendix S1: Individual and contextual data

This appendix details the individual and contextual characteristics included in the study. All variables correspond to maternal characteristics except when otherwise is indicated.

Individual data

Sociodemographic traits

Maternal and paternal age, maternal and paternal schooling (<7 years of school education), cohabitation with current partner, and self-reported ancestry (Amerindian, Latin European, Non-Latin European, Arab, African, Asian, and Jewish).

Reproductive history, biological and anthropometric characteristics

Number of gestation, change of partner between current and previous pregnancy, previous multiple pregnancy, body mass index at the beginning of pregnancy, blood ABO type and Rh factor, and newborn sex.

Prenatal care

Few prenatal visits (<5), place of prenatal visits (hospital or primary healthcare center), ultrasound examination (in the first and second trimester), serologic testing (*e. g.* toxoplasmosis test), papanicolaou test, vaccination during pregnancy (in the first and second trimester), and dentist consultation during pregnancy.

Stressful situations

Divorce, lack of family support, death of a family member, changing residence, job loss, domestic violence, and other criminal situations.

Diseases and health complications

The following illnesses and complications prior to or during pregnancy were included: asthma, chronic hypertension, difficulty in conceiving, vaginal bleeding (in the first and second trimester), vaginal discharge, urinary tract infection -UTI- (UTI, symptomatic cystitis, or pyelonephritis), Chagas' disease, toxoplasmosis, anemia, dental treatment (*e. g.* dental cavities and extractions), periodontal disease, sexually transmitted diseases, lupus, other autoimmune diseases, type 1 and 2 *diabetes mellitus*, arthritis, psychological disorder, fibroid / myoma, surgery before pregnancy, and major medical procedure during pregnancy (*e. g.* blood transfusion).

Medications and supplements

The following were considered: iron, magnesium, or folic acid supplement; vitamins; anemia medication; UTI treatment; and medication intake before pregnancy.

Habits and activities

Tobacco smoking before and during pregnancy, passive smoking; alcohol consumption before and during pregnancy; coffee, tea, or mate intake; illicit drugs use; physical activity before and during pregnancy; sexual activity during the last month of pregnancy; hypocaloric diet during pregnancy; and work during pregnancy.

Imputation of individual level variables

Variables with more than 20% missing data were discarded. On the remaining variables we imputed the missing data using decision trees based on resampling aggregation (Kuhn, 2008).

Contextual data

Proportion of neighbourhood households without Unsatisfied Basic Needs

To estimate neighbourhood socioeconomic status, the Unsatisfied Basic Needs (UBN) index from the Argentine 2010 national census was used (Instituto Nacional de Estadística y Censos, 2010). A housing unit has UBN when at least one of the following conditions is present (Feres and Mancero, 2001; Instituto Nacional de Estadística y Censos, 2010): the family resides in a pension, tenancy, hotel, precarious dwelling, or facility not intended for housing purposes; the house does not have a toilet; there are more than three people per room; there is at least one child, 6 to 12 years old, who does not attend to school; in the household, four or more people exist per employed family member and its head member has not completed third grade of primary school (<4 years of schooling).

The percentage of households without UBN linked to each maternal domicile was determined using the census radii. The census radius is a censal unit which represents the smallest territorial entity with available data and comprises 100 to 300 dwellings on average (Instituto Nacional de Estadística y Censos, 2021). The census radii close to each maternal domicile were identified by superimposing 100m Euclidean buffers around the domiciles with a specified street name and number and 500m Euclidean buffers around the estimated vicinity centromere of domiciles that only specified the neighbourhood. For each maternal domicile, the percentage of households without UBN was weighted with the percentage of the overlapping surface of the census radius in the defined buffer. R sf package was used (Pebesma, 2018).

Urban conglomerate

To consider the heterogeneity between urban and rural populations, the general population was stratified according to the number of inhabitants. To do this, the 2010 National Census' centromeres of the census radii were grouped using the Density Based Clustering of Applications with Noise (DBSCAN) algorithm (Instituto Nacional de Estadística y Censos, 2010; Hahsler *et al.*, 2019). We used a minimum number of points equal to 2 and an epsilon of 1000m, which was estimated by analysing the distribution of 2-nearest neighbour distances. The

number of inhabitants of each group was calculated by adding the number of inhabitants of the census radii that compose it. Finally, we defined two population strata based on the group of census radii with the largest number of inhabitants (791649), which contained 47.7% of the general studied population and included San Miguel de Tucumán, the capital city of Tucumán province, and surrounding cities. R packages sf and dbscan were used (Pebesma, 2018; Hahsler *et al.*, 2019).

References

Feres JC and Mancero X (2001) El método de las necesidades básicas insatisfechas (NBI) y sus aplicaciones en América Latina. Comisión Económica para América Latina y el Caribe - Organización de las Naciones Unidas, Santiago de Chile.

Hahsler M, Piekenbrock M and Doran D (2019) dbscan: Fast density-based clustering with R. J Stat Soft 91:1-30.

Kuhn M (2008) Building predictive models in R using the caret package. J Stat Soft 28:1-26.

Pebesma EJ (2018) Simple features for R: standardized support for spatial vector data. R J 10:439.

Internet Resources

Instituto Nacional de Estadística y Censos (2010) Censo Nacional de Población, Hogares y Viviendas 2010, https://www.indec.gob.ar/indec/web/Nivel4-Tema-2-41-135_(accessed 14 January 2023).

Instituto Nacional de Estadística y Censos (2021) Unidades Geoestadísticas, https://geoservicios.indec.gov.ar/codgeo/index.php?pagina=definiciones (accessed 14 January 2023).
