# Supplementary material for: Gene-environment interactions and preterm birth predictors: A Bayesian network approach
Source: Genet Mol Biol. 2024 Jan 19;46(4):e20230090. doi: 10.1590/1678-4685-GMB-2023-0090 (PMC10804443; doi:10.1590/1678-4685-GMB-2023-0090)
Supplement: Appendix S2 - [file 1415-4757-GMB-46-4-e20230090-s2.pdf]

Genetics and Molecular Biology
https://doi.org/10.1590/1678-4685-GMB-2023-0090

Supplementary Material to "Gene-environment interactions and preterm birth predictors: A Bayesian network approach"

Appendix S2 - Sequencing and variant calling of newborn genes

Sequencing

DNA samples were obtained from placental blood cord. We selected the *COL4A3, KCNN3, F3, PON1*, and *CRHR1* genes to be sequenced because in a previous study, conducted in the same population, we identified variants associated with preterm birth (Gimenez *et al.*, 2017). Custom NGS panel was designed using Ion Ampliseq™ Designer, pipeline v6.1.3 (Thermo Fisher Scientific), targeting coding regions plus 20bp of intron-exon junction of selected genes. Average target coverage was 98.1%. Twenty nanograms of each gDNA sample were used for target PCR-based enrichment. Libraries were prepared according to the Ion AmpliSeq™ Library kit 2.0 user guide (Thermo Fisher Scientific). Samples were barcoded with Ion Xpress™ Barcode Adapters kit (Thermo Fisher Scientific). Libraries were purified with Agencourt AMPure XP kit (Beckman Coulter) and then quantified using the Ion Library TaqMan™ Quantification Kit (Thermo Fisher Scientific), followed by dilution to the same concentration (100pM), pooled in equal volume aliquots, and then loaded on to the Ion Chef™ Instrument (Thermo Fisher Scientific) for emulsion PCR, enrichment, and loading onto the Ion S5 540 chip. Sequencing was performed on the GeneStudio™ S5 System (Thermo Fisher Scientific).

Variant calling

Raw sequencing data was analysed using Torrent Suite Software version 5.10 (Thermo Fisher Scientific). Primary analysis included signal processing, base calling, demultiplexing, read alignment to human genome 19 reference (Genome Reference Consortium GRCh37), and quality control of mapping quality. Variant calling was done with Torrent Variant Caller

(Thermo Fisher Scientific) software using suggested parametrization. Homozygote reference genotypes across samples was completed using JVarkit software (Lindenbaum, 2015). Variants with more than two alleles were excluded using bcftools software (Danecek *et al.*, 2021). Also variants with genotype quality less than 15 or deep coverage less than 35X were excluded per sample. Then, using PLINK software, variants with minimum allele frequency <5%, genotypes missing >10%, or deviations from Hardy–Weinberg equilibrium ($p < 0.001$) were excluded (Purcell *et al.*, 2007). Samples with more than 10% of missing genotype were excluded. The missing genotypes in the remaining samples (58 of 7410 genotypes) were imputed using Beagle 5.1 software (Browning *et al.*, 2018). Variant Effect Predictor software was used for variant annotation (McLaren *et al.*, 2016).

References

Browning BL, Zhou Y and Browning SR (2018) A one-penny imputed genome from next-generation reference panels. Am J Hum Genet 103:338-348.

Danecek P, Bonfield JK, Liddle J, Marshall J, Ohan V, Pollard MO, Whitwham A, Keane T, McCarthy SA, Davies RM *et al.* (2021) Twelve years of SAMtools and BCFtools. Gigascience 10:giab008.

Gimenez LG, Momany AM, Poletta FA, Krupitzki HB, Gili JA, Busch TD, Saleme C, Cosentino VR, Pawluk MS, Campaña H *et al.* (2017) Association of candidate gene polymorphisms with clinical subtypes of preterm birth in a Latin American population. Pediatr Res 82:554-559.

McLaren W, Gil L, Hunt SE, Riat HS, Ritchie GR, Thormann A, Flicek P and Cunningham F (2016) The ensembl variant effect predictor. Genome Biol 17:1-14.

Purcell S, Neale B, Todd-Brown K, Thomas L, Ferreira MA, Bender D, Maller J, Sklar P, de Bakker PI, Daly MJ *et al.* (2007) PLINK: A tool set for whole-genome association and population-based linkage analyses. Am J Hum Genet 81:559-575.

Internet Resources

Lindenbaum P (2015) JVarkit: Java-based utilities for Bioinformatics, https://doi.org/10.6084/m9.figshare.1425030.v1 (accessed 14 January 2023).
