# Supplementary material for: Gene-environment interactions and preterm birth predictors: A Bayesian network approach
Source: Genet Mol Biol. 2024 Jan 19;46(4):e20230090. doi: 10.1590/1678-4685-GMB-2023-0090 (PMC10804443; doi:10.1590/1678-4685-GMB-2023-0090)
Supplement: Table S1 - [file 1415-4757-GMB-46-4-e20230090-s3.pdf]

Genetics and Molecular Biology
https://doi.org/10.1590/1678-4685-GMB-2023-0090

# Supplementary Material to "Gene-environment interactions and preterm birth predictors: A Bayesian network approach"

**Table S1 -** Potential edge directions excluded from Bayesian network structure learning. Abbreviations: NSES, neighbourhood socioeconomic status.

| From | To |
|---|---|
| High body mass index at the beginning of pregnancy | Low body mass index at the beginning of pregnancy |
| Low body mass index at the beginning of pregnancy | High body mass index at the beginning of pregnancy |
| High maternal age | Low maternal age |
| Low maternal age | High maternal age |
| High number of gestation | Low number of gestation |
| Low number of gestation | High number of gestation |
| High NSES | Low NSES |
| Low NSES | High NSES |
| Preterm birth | Maternal blood ABO group A |
| Preterm birth | High body mass index at the beginning of pregnancy |
| Preterm birth | Low body mass index at the beginning of pregnancy |
| Preterm birth | High maternal age |
| Preterm birth | Low maternal age |
| Preterm birth | High number of gestation |
| Preterm birth | Low number of gestation |
| Preterm birth | Few prenatal visits |
| Preterm birth | Anemia |
| Preterm birth | Toxoplasmosis |
| Preterm birth | Sexual activity during the last month of pregnancy |
| Preterm birth | rs11680670 (*COL4A3*) |
| Preterm birth | rs2074351 (*PON1*) |

| From | To |
|---|---|
| Preterm birth | rs73993878 (*COL4A3*) |
| Preterm birth | rs12621551 (*COL4A3*) |
| Preterm birth | rs8073146 (*CRHR1*) |
| Preterm birth | rs4845397 (*KCNN3*) |
| High number of gestation | Maternal blood ABO group A |
| High number of gestation | High body mass index at the beginning of pregnancy |
| High number of gestation | Low body mass index at the beginning of pregnancy |
| High number of gestation | High maternal age |
| High number of gestation | Low maternal age |
| Low number of gestation | Maternal blood ABO group A |
| Low number of gestation | High body mass index at the beginning of pregnancy |
| Low number of gestation | Low body mass index at the beginning of pregnancy |
| Low number of gestation | High maternal age |
| Low number of gestation | Low maternal age |
| Few prenatal visits | Maternal blood ABO group A |
| Few prenatal visits | High body mass index at the beginning of pregnancy |
| Few prenatal visits | Low body mass index at the beginning of pregnancy |
| Few prenatal visits | High maternal age |
| Few prenatal visits | Low maternal age |
| Anemia | Maternal blood ABO group A |
| Anemia | High body mass index at the beginning of pregnancy |
| Anemia | Low body mass index at the beginning of pregnancy |
| Anemia | High maternal age |
| Anemia | Low maternal age |
| Toxoplasmosis | Maternal blood ABO group A |
| Toxoplasmosis | High body mass index at the beginning of pregnancy |
| Toxoplasmosis | Low body mass index at the beginning of pregnancy |
| Toxoplasmosis | High maternal age |

| From | To |
|---|---|
| Toxoplasmosis | Low maternal age |
| Sexual activity during the last month of pregnancy | Maternal blood ABO group A |
| Sexual activity during the last month of pregnancy | High body mass index at the beginning of pregnancy |
| Sexual activity during the last month of pregnancy | Low body mass index at the beginning of pregnancy |
| Sexual activity during the last month of pregnancy | High maternal age |
| Sexual activity during the last month of pregnancy | Low maternal age |
| rs11680670 (*COL4A3*) | Maternal blood ABO group A |
| rs11680670 (*COL4A3*) | High body mass index at the beginning of pregnancy |
| rs11680670 (*COL4A3*) | Low body mass index at the beginning of pregnancy |
| rs11680670 (*COL4A3*) | High maternal age |
| rs11680670 (*COL4A3*) | Low maternal age |
| rs2074351 (*PON1*) | Maternal blood ABO group A |
| rs2074351 (*PON1*) | High body mass index at the beginning of pregnancy |
| rs2074351 (*PON1*) | Low body mass index at the beginning of pregnancy |
| rs2074351 (*PON1*) | High maternal age |
| rs2074351 (*PON1*) | Low maternal age |
| rs73993878 (*COL4A3*) | Maternal blood ABO group A |
| rs73993878 (*COL4A3*) | High body mass index at the beginning of pregnancy |
| rs73993878 (*COL4A3*) | Low body mass index at the beginning of pregnancy |
| rs73993878 (*COL4A3*) | High maternal age |
| rs73993878 (*COL4A3*) | Low maternal age |
| rs12621551 (*COL4A3*) | Maternal blood ABO group A |
| rs12621551 (*COL4A3*) | High body mass index at the beginning of pregnancy |
| rs12621551 (*COL4A3*) | Low body mass index at the beginning of pregnancy |
| rs12621551 (*COL4A3*) | High maternal age |
| rs12621551 (*COL4A3*) | Low maternal age |
| rs8073146 (*CRHR1*) | Maternal blood ABO group A |
| rs8073146 (*CRHR1*) | High body mass index at the beginning of pregnancy |

| From | To |
|---|---|
| rs8073146 (*CRHR1*) | Low body mass index at the beginning of pregnancy |
| rs8073146 (*CRHR1*) | High maternal age |
| rs8073146 (*CRHR1*) | Low maternal age |
| rs4845397 (*KCNN3*) | Maternal blood ABO group A |
| rs4845397 (*KCNN3*) | High body mass index at the beginning of pregnancy |
| rs4845397 (*KCNN3*) | Low body mass index at the beginning of pregnancy |
| rs4845397 (*KCNN3*) | High maternal age |
| rs4845397 (*KCNN3*) | Low maternal age |
| High body mass index at the beginning of pregnancy | High number of gestation |
| High body mass index at the beginning of pregnancy | Low number of gestation |
| Low body mass index at the beginning of pregnancy | High number of gestation |
| Low body mass index at the beginning of pregnancy | Low number of gestation |
| Anemia | Few prenatal visits |
| Anemia | rs11680670 (*COL4A3*) |
| Anemia | rs2074351 (*PON1*) |
| Anemia | rs73993878 (*COL4A3*) |
| Anemia | rs12621551 (*COL4A3*) |
| Anemia | rs8073146 (*CRHR1*) |
| Anemia | rs4845397 (*KCNN3*) |
| Toxoplasmosis | Few prenatal visits |
| Toxoplasmosis | rs11680670 (*COL4A3*) |
| Toxoplasmosis | rs2074351 (*PON1*) |
| Toxoplasmosis | rs73993878 (*COL4A3*) |
| Toxoplasmosis | rs12621551 (*COL4A3*) |
| Toxoplasmosis | rs8073146 (*CRHR1*) |
| Toxoplasmosis | rs4845397 (*KCNN3*) |
| Sexual activity during the last month of pregnancy | Few prenatal visits |
| Sexual activity during the last month of pregnancy | rs11680670 (*COL4A3*) |

| From | To |
|---|---|
| Sexual activity during the last month of pregnancy | rs2074351 (*PON1*) |
| Sexual activity during the last month of pregnancy | rs73993878 (*COL4A3*) |
| Sexual activity during the last month of pregnancy | rs12621551 (*COL4A3*) |
| Sexual activity during the last month of pregnancy | rs8073146 (*CRHR1*) |
| Sexual activity during the last month of pregnancy | rs4845397 (*KCNN3*) |
| Anemia | High number of gestation |
| Anemia | Low number of gestation |
| Toxoplasmosis | High number of gestation |
| Toxoplasmosis | Low number of gestation |
| Sexual activity during the last month of pregnancy | High number of gestation |
| Sexual activity during the last month of pregnancy | Low number of gestation |
| Few prenatal visits | High number of gestation |
| Few prenatal visits | Low number of gestation |
| rs11680670 (*COL4A3*) | High number of gestation |
| rs11680670 (*COL4A3*) | Low number of gestation |
| rs2074351 (*PON1*) | High number of gestation |
| rs2074351 (*PON1*) | Low number of gestation |
| rs73993878 (*COL4A3*) | High number of gestation |
| rs73993878 (*COL4A3*) | Low number of gestation |
| rs12621551 (*COL4A3*) | High number of gestation |
| rs12621551 (*COL4A3*) | Low number of gestation |
| rs8073146 (*CRHR1*) | High number of gestation |
| rs8073146 (*CRHR1*) | Low number of gestation |
| rs4845397 (*KCNN3*) | High number of gestation |
| rs4845397 (*KCNN3*) | Low number of gestation |
| Few prenatal visits | rs11680670 (*COL4A3*) |
| Few prenatal visits | rs2074351 (*PON1*) |
| Few prenatal visits | rs73993878 (*COL4A3*) |

| From | To |
|---|---|
| Few prenatal visits | rs12621551 (*COL4A3*) |
| Few prenatal visits | rs8073146 (*CRHR1*) |
| Few prenatal visits | rs4845397 (*KCNN3*) |
| Preterm birth | High NSES |
| Preterm birth | Low NSES |
| Maternal blood ABO group A | High NSES |
| Maternal blood ABO group A | Low NSES |
| High body mass index at the beginning of pregnancy | High NSES |
| High body mass index at the beginning of pregnancy | Low NSES |
| Low body mass index at the beginning of pregnancy | High NSES |
| Low body mass index at the beginning of pregnancy | Low NSES |
| High maternal age | High NSES |
| High maternal age | Low NSES |
| Low maternal age | High NSES |
| Low maternal age | Low NSES |
| High number of gestation | High NSES |
| High number of gestation | Low NSES |
| Low number of gestation | High NSES |
| Low number of gestation | Low NSES |
| Few prenatal visits | High NSES |
| Few prenatal visits | Low NSES |
| Anemia | High NSES |
| Anemia | Low NSES |
| Toxoplasmosis | High NSES |
| Toxoplasmosis | Low NSES |
| Sexual activity during the last month of pregnancy | High NSES |
| Sexual activity during the last month of pregnancy | Low NSES |
| rs11680670 (*COL4A3*) | High NSES |

| **From** | **To** |
| --- | --- |
| rs11680670 (*COL4A3*) | Low NSES |
| rs2074351 (*PON1*) | High NSES |
| rs2074351 (*PON1*) | Low NSES |
| rs73993878 (*COL4A3*) | High NSES |
| rs73993878 (*COL4A3*) | Low NSES |
| rs12621551 (*COL4A3*) | High NSES |
| rs12621551 (*COL4A3*) | Low NSES |
| rs8073146 (*CRHR1*) | High NSES |
| rs8073146 (*CRHR1*) | Low NSES |
| rs4845397 (*KCNN3*) | High NSES |
| rs4845397 (*KCNN3*) | Low NSES |
