# Supplementary material for: Gene-environment interactions and preterm birth predictors: A Bayesian network approach
Source: Genet Mol Biol. 2024 Jan 19;46(4):e20230090. doi: 10.1590/1678-4685-GMB-2023-0090 (PMC10804443; doi:10.1590/1678-4685-GMB-2023-0090)
Supplement: Table S2 - [file 1415-4757-GMB-46-4-e20230090-s4.pdf]

Genetics and Molecular Biology
https://doi.org/10.1590/1678-4685-GMB-2023-0090

Supplementary Material to "Gene-environment interactions and preterm birth predictors: A Bayesian network approach"

Table S2 - Regression models tested with the interaction between rs2074351 (*PON1*) and NSES for toxoplasmosis. Abbreviations: CI, confidence interval; NSES, neighbourhood socioeconomic status.

Specific covariate	Variable	Odds ratio (95% CI)	P Value
Without covariates	Low NSES	0.31 (0.06 - 1.52)	0.1503
	rs2074351 (*PON1*)	0.83 (0.30 - 2.32)	0.7225
	High NSES	0.52 (0.12 - 2.18)	0.3690
	Low NSES : rs2074351 (*PON1*)	12.51 (1.71 - 91.36)	0.0127
	rs2074351 (*PON1*) : High NSES	1.50 (0.22 - 10.04)	0.6747
Self-reported ancestry	Low NSES	0.32 (0.06 - 1.61)	0.1667
	rs2074351 (*PON1*)	0.82 (0.28 - 2.36)	0.7060
	High NSES	0.50 (0.11 - 2.25)	0.3673
	Non-native ancestors	0.37 (0.04 - 3.62)	0.3932
	Low NSES : rs2074351 (*PON1*)	10.85 (1.44 - 82.00)	0.0208
	rs2074351 (*PON1*) : High NSES	1.47 (0.22 - 9.85)	0.6916
	rs2074351 (*PON1*) : Non-native ancestors	1.02 (0.06 - 16.33)	0.9897
	Low NSES : Non-native ancestors	2.48 (0.03 - 243.76)	0.6975
	High NSES : Non-native ancestors	2.19 (0.13 - 35.76)	0.5812
Low maternal age	Low NSES	0.41 (0.06 - 2.73)	0.3538
	rs2074351 (*PON1*)	0.74 (0.23 - 2.42)	0.6244
	High NSES	0.71 (0.14 - 3.52)	0.6750
	Low maternal age	3.54 (0.73 - 17.05)	0.1157
	Low NSES : rs2074351 (*PON1*)	13.50 (1.66 - 109.67)	0.0149

Specific covariate	Variable	Odds ratio (95% CI)	P Value
	rs2074351 (*PON1*) : High NSES	1.67 (0.24 - 11.53)	0.6010
	rs2074351 (*PON1*) : Low maternal age	0.93 (0.16 - 5.31)	0.9321
	Low NSES : Low maternal age	0.34 (0.04 - 2.92)	0.3273
	High NSES : Low maternal age	0.29 (0.03 - 2.49)	0.2615
High maternal age	Low NSES	0.29 (0.05 - 1.60)	0.1566
	rs2074351 (*PON1*)	0.91 (0.29 - 2.81)	0.8643
	High NSES	0.51 (0.11 - 2.44)	0.3984
	High maternal age	1.47 (0.29 - 7.42)	0.6413
	Low NSES : rs2074351 (*PON1*)	11.79 (1.61 - 86.17)	0.0150
	rs2074351 (*PON1*) : High NSES	1.54 (0.23 - 10.40)	0.6589
	rs2074351 (*PON1*) : High maternal age	0.66 (0.11 - 3.84)	0.6454
	Low NSES : High maternal age	1.27 (0.16 - 9.97)	0.8211
	High NSES : High maternal age	1.01 (0.12 - 8.74)	0.9894
Tobacco smoking before pregnancy	Low NSES	0.33 (0.05 - 2.24)	0.2578
	rs2074351 (*PON1*)	1.71 (0.50 - 5.89)	0.3943
	High NSES	0.51 (0.08 - 3.09)	0.4656
	Tobacco smoking before pregnancy	2.57 (0.63 - 10.40)	0.1866
	Low NSES : rs2074351 (*PON1*)	10.25 (1.29 - 81.49)	0.0278
	rs2074351 (*PON1*) : High NSES	1.53 (0.21 - 11.15)	0.6727
	rs2074351 (*PON1*) : Tobacco smoking before pregnancy	0.17 (0.03 - 0.86)	0.0327
	Low NSES : Tobacco smoking before pregnancy	0.72 (0.09 - 5.71)	0.7574
	High NSES : Tobacco smoking before pregnancy	0.75 (0.10 - 5.52)	0.7816
Tobacco smoking during pregnancy	Low NSES	0.32 (0.06 - 1.71)	0.1835
	rs2074351 (*PON1*)	1.22 (0.37 - 3.97)	0.7460

Specific covariate	Variable	Odds ratio (95% CI)	P Value
	High NSES	0.44 (0.09 - 2.20)	0.3197
	Tobacco smoking during pregnancy	1.17 (0.24 - 5.81)	0.8465
	Low NSES : rs2074351 (*PON1*)	8.78 (1.13 - 68.13)	0.0377
	rs2074351 (*PON1*) : High NSES	1.33 (0.19 - 9.22)	0.7705
	rs2074351 (*PON1*) : Tobacco smoking during pregnancy	0.39 (0.06 - 2.47)	0.3175
	Low NSES : Tobacco smoking during pregnancy	1.29 (0.08 - 20.66)	0.8575
	High NSES : Tobacco smoking during pregnancy	1.68 (0.19 - 14.44)	0.6388
Passive smoking	Low NSES	0.26 (0.05 - 1.41)	0.1181
	rs2074351 (*PON1*)	0.77 (0.23 - 2.55)	0.6711
	High NSES	0.62 (0.12 - 3.17)	0.5653
	Passive smoking	0.53 (0.12 - 2.27)	0.3902
	Low NSES : rs2074351 (*PON1*)	9.53 (1.29 - 70.33)	0.0271
	rs2074351 (*PON1*) : High NSES	1.24 (0.18 - 8.55)	0.8264
	rs2074351 (*PON1*) : Passive smoking	1.50 (0.29 - 7.75)	0.6298
	Low NSES : Passive smoking	2.26 (0.32 - 16.01)	0.4130
	High NSES : Passive smoking	0.92 (0.12 - 7.10)	0.9382
Alcohol intake before pregnancy	Low NSES	0.37 (0.07 - 1.84)	0.2228
	rs2074351 (*PON1*)	0.96 (0.32 - 2.88)	0.9350
	High NSES	0.49 (0.11 - 2.18)	0.3492
	Alcohol intake before pregnancy	0.30 (0.04 - 2.38)	0.2565
	Low NSES : rs2074351 (*PON1*)	13.04 (1.60 - 106.46)	0.0165
	rs2074351 (*PON1*) : High NSES	1.30 (0.19 - 8.68)	0.7883
	rs2074351 (*PON1*) : Alcohol intake before pregnancy	0.94 (0.10 - 9.18)	0.9593
	Low NSES : Alcohol intake before pregnancy	0.45 (0.03 - 7.00)	0.5709
	High NSES : Alcohol intake before pregnancy	2.18 (0.18 - 26.87)	0.5420

Specific covariate	Variable	Odds ratio (95% CI)	P Value
Alcohol intake during pregnancy	Low NSES	0.34 (0.07 - 1.68)	0.1869
	rs2074351 (*PON1*)	0.92 (0.32 - 2.63)	0.8799
	High NSES	0.47 (0.11 - 2.06)	0.3148
	Alcohol intake during pregnancy	1.30 (0.07 - 25.29)	0.8604
	Low NSES : rs2074351 (*PON1*)	11.65 (1.60 - 84.61)	0.0152
	rs2074351 (*PON1*) : High NSES	1.51 (0.22 - 10.23)	0.6756
	rs2074351 (*PON1*) : Alcohol intake during pregnancy	0.38 (0.02 - 6.63)	0.5062
	Low NSES : Alcohol intake during pregnancy	0.77 (0.03 - 19.09)	0.8754
	High NSES : Alcohol intake during pregnancy	2.61 (0.10 - 70.38)	0.5680
Male newborn sex	Low NSES	0.23 (0.03 - 1.67)	0.1444
	rs2074351 (*PON1*)	1.17 (0.34 - 4.00)	0.8016
	High NSES	0.13 (0.01 - 1.68)	0.1181
	Male newborn sex	2.19 (0.52 - 9.24)	0.2849
	Low NSES : rs2074351 (*PON1*)	13.89 (1.80 - 107.49)	0.0117
	rs2074351 (*PON1*) : High NSES	3.64 (0.38 - 34.94)	0.2623
	rs2074351 (*PON1*) : Male newborn sex	0.38 (0.07 - 2.16)	0.2736
	Low NSES : Male newborn sex	1.43 (0.20 - 10.28)	0.7237
	High NSES : Male newborn sex	3.08 (0.33 - 28.99)	0.3262
Low maternal schooling (<7 years of school education)	Low NSES	0.18 (0.03 - 1.19)	0.0746
	rs2074351 (*PON1*)	0.59 (0.19 - 1.82)	0.3589
	High NSES	0.54 (0.12 - 2.39)	0.4184
	Low maternal schooling	0.34 (0.05 - 2.38)	0.2782
	Low NSES : rs2074351 (*PON1*)	18.61 (1.98 - 174.55)	0.0105
	rs2074351 (*PON1*) : High NSES	1.57 (0.23 - 10.69)	0.6446
	rs2074351 (*PON1*) : Low maternal schooling	5.38 (0.55 - 52.41)	0.1471

Specific covariate	Variable	Odds ratio (95% CI)	P Value
	Low NSES : Low maternal schooling	12.69 (0.52 - 308.91)	0.1188
	High NSES : Low maternal schooling	0.78 (0.06 - 10.38)	0.8533
Anemia	Low NSES	0.48 (0.09 - 2.64)	0.3984
	rs2074351 (*PON1*)	0.33 (0.08 - 1.48)	0.1496
	High NSES	1.39 (0.26 - 7.46)	0.7025
	Anemia	1.61 (0.41 - 6.30)	0.4911
	Low NSES : rs2074351 (*PON1*)	17.78 (2.25 - 140.69)	0.0064
	rs2074351 (*PON1*) : High NSES	2.00 (0.26 - 15.37)	0.5039
	rs2074351 (*PON1*) : Anemia	3.48 (0.64 - 18.77)	0.1475
	Low NSES : Anemia	0.38 (0.05 - 3.05)	0.3645
	High NSES : Anemia	0.11 (0.01 - 0.89)	0.0380
Low body mass index at the beginning of pregnancy	Low NSES	0.41 (0.08 - 2.04)	0.2758
	rs2074351 (*PON1*)	0.48 (0.14 - 1.62)	0.2347
	High NSES	0.61 (0.14 - 2.67)	0.5137
	Low body mass index at the beginning of pregnancy	0.92 (0.16 - 5.21)	0.9275
	Low NSES : rs2074351 (*PON1*)	14.91 (1.95 - 114.09)	0.0093
	rs2074351 (*PON1*) : High NSES	1.86 (0.26 - 13.29)	0.5365
	rs2074351 (*PON1*) : Low body mass index at the beginning of pregnancy	4.13 (0.59 - 29.15)	0.1549
	Low NSES : Low body mass index at the beginning of pregnancy	0.33 (0.03 - 3.62)	0.3632
	High NSES : Low body mass index at the beginning of pregnancy	0.48 (0.05 - 4.38)	0.5126
High body mass index at the beginning of pregnancy	Low NSES	0.45 (0.09 - 2.29)	0.3337
	rs2074351 (*PON1*)	0.94 (0.29 - 3.03)	0.9203
	High NSES	0.68 (0.15 - 3.19)	0.6295

Specific covariate	Variable	Odds ratio (95% CI)	P Value
	High body mass index at the beginning of pregnancy	1.65 (0.40 - 6.86)	0.4901
	Low NSES : rs2074351 (*PON1*)	13.50 (1.70 - 107.17)	0.0138
	rs2074351 (*PON1*) : High NSES	1.37 (0.20 - 9.19)	0.7463
	rs2074351 (*PON1*) : High body mass index at the beginning of pregnancy	0.77 (0.12 - 4.95)	0.7823
	Low NSES : High body mass index at the beginning of pregnancy	0.26 (0.03 - 2.57)	0.2500
	High NSES : High body mass index at the beginning of pregnancy	0.45 (0.04 - 4.69)	0.5006
Maternal blood ABO group A	Low NSES	0.14 (0.02 - 1.14)	0.0663
	rs2074351 (*PON1*)	1.21 (0.38 - 3.85)	0.7446
	High NSES	0.16 (0.02 - 1.41)	0.1002
	Maternal blood ABO group A	1.63 (0.34 - 7.84)	0.5403
	Low NSES : rs2074351 (*PON1*)	16.62 (1.73 - 159.52)	0.0148
	rs2074351 (*PON1*) : High NSES	2.72 (0.31 - 24.09)	0.3682
	rs2074351 (*PON1*) : Maternal blood ABO group A	0.31 (0.05 - 1.86)	0.1991
	Low NSES : Maternal blood ABO group A	5.53 (0.53 - 57.33)	0.1516
	High NSES : Maternal blood ABO group A	4.55 (0.56 - 36.91)	0.1558
High number of gestation	Low NSES	0.27 (0.05 - 1.32)	0.1061
	rs2074351 (*PON1*)	0.60 (0.20 - 1.85)	0.3761
	High NSES	0.42 (0.09 - 1.95)	0.2702
	High number of gestation	0.30 (0.05 - 1.62)	0.1605
	Low NSES : rs2074351 (*PON1*)	13.12 (1.63 - 105.28)	0.0154
	rs2074351 (*PON1*) : High NSES	1.32 (0.19 - 9.02)	0.7797
	rs2074351 (*PON1*) : High number of gestation	3.88 (0.55 - 27.54)	0.1758
	Low NSES : High number of gestation	1.13 (0.11 - 12.00)	0.9183
	High NSES : High number of gestation	2.85 (0.34 - 23.93)	0.3345

Specific covariate	Variable	Odds ratio (95% CI)	P Value
Low number of gestation	Low NSES	0.86 (0.14 - 5.29)	0.8746
	rs2074351 (*PON1*)	0.81 (0.23 - 2.88)	0.7496
	High NSES	0.92 (0.18 - 4.78)	0.9180
	Low number of gestation	3.29 (0.84 - 12.87)	0.0872
	Low NSES : rs2074351 (*PON1*)	9.54 (1.27 - 71.53)	0.0282
	rs2074351 (*PON1*) : High NSES	1.41 (0.21 - 9.56)	0.7220
	rs2074351 (*PON1*) : Low number of gestation	1.16 (0.23 - 5.78)	0.8524
	Low NSES : Low number of gestation	0.12 (0.02 - 0.89)	0.0379
	High NSES : Low number of gestation	0.27 (0.04 - 1.84)	0.1804
Sexual activity during the last month of pregnancy	Low NSES	0.33 (0.06 - 1.85)	0.2071
	rs2074351 (*PON1*)	1.23 (0.38 - 3.96)	0.7293
	High NSES	0.38 (0.07 - 2.07)	0.2648
	Sexual activity during the last month of pregnancy	1.90 (0.47 - 7.67)	0.3663
	Low NSES : rs2074351 (*PON1*)	11.87 (1.62 - 87.04)	0.0149
	rs2074351 (*PON1*) : High NSES	1.35 (0.18 - 10.25)	0.7705
	rs2074351 (*PON1*) : Sexual activity during the last month of pregnancy	0.30 (0.05 - 1.68)	0.1707
	Low NSES : Sexual activity during the last month of pregnancy	1.10 (0.15 - 8.29)	0.9227
	High NSES : Sexual activity during the last month of pregnancy	6.09 (0.59 - 63.31)	0.1302
Few prenatal visits (<5)	Low NSES	0.34 (0.06 - 2.05)	0.2408
	rs2074351 (*PON1*)	0.49 (0.14 - 1.74)	0.2695
	High NSES	0.29 (0.06 - 1.47)	0.1354
	Few prenatal visits (<5)	0.34 (0.09 - 1.34)	0.1247
	Low NSES : rs2074351 (*PON1*)	10.42 (1.42 - 76.56)	0.0213

Specific covariate	Variable	Odds ratio (95% CI)	P Value
	rs2074351 (*PON1*) : High NSES	1.14 (0.15 - 8.54)	0.9000
	rs2074351 (*PON1*) : Few prenatal visits (<5)	3.36 (0.68 - 16.51)	0.1365
	Low NSES : Few prenatal visits (<5)	1.12 (0.16 - 7.63)	0.9116
	High NSES : Few prenatal visits (<5)	4.46 (0.60 - 33.07)	0.1433
Live in a largest urban conglomerate	Low NSES	0.37 (0.06 - 2.38)	0.2985
	rs2074351 (*PON1*)	0.47 (0.10 - 2.21)	0.3365
	High NSES	0.52 (0.06 - 4.26)	0.5419
	Live in a largest urban conglomerate	0.68 (0.16 - 2.93)	0.6090
	Low NSES : rs2074351 (*PON1*)	17.19 (1.95 - 151.41)	0.0104
	rs2074351 (*PON1*) : High NSES	1.43 (0.21 - 9.59)	0.7105
	rs2074351 (*PON1*) : Live in a largest urban conglomerate	2.44 (0.44 - 13.52)	0.3068
	Low NSES : Live in a largest urban conglomerate	0.37 (0.05 - 2.90)	0.3417
	High NSES : Live in a largest urban conglomerate	1.06 (0.13 - 8.31)	0.9566
Domicile accuracy at the neighborhood level	Low NSES	0.15 (0.01 - 2.07)	0.1572
	rs2074351 (*PON1*)	1.03 (0.29 - 3.63)	0.9684
	High NSES	0.57 (0.12 - 2.76)	0.4848
	Domicile accuracy at the neighborhood level	0.78 (0.20 - 3.05)	0.7192
	Low NSES : rs2074351 (*PON1*)	14.68 (1.75 - 123.46)	0.0134
	rs2074351 (*PON1*) : High NSES	1.38 (0.20 - 9.62)	0.7436
	rs2074351 (*PON1*) : Domicile accuracy at the neighborhood level	0.58 (0.10 - 3.33)	0.5435
	Low NSES : Domicile accuracy at the neighborhood level	2.82 (0.24 - 32.72)	0.4077
	High NSES : Domicile accuracy at the neighborhood level	0.65 (0.07 - 6.04)	0.7049
rs8073146 (*CRHR1*)	Low NSES	0.34 (0.07 - 1.68)	0.1832

Specific covariate	Variable	Odds ratio (95% CI)	P Value
	rs2074351 (*PON1*)	0.98 (0.31 - 3.06)	0.9725
	High NSES	0.69 (0.15 - 3.25)	0.6350
	rs8073146 (*CRHR1*)	0.99 (0.20 - 4.88)	0.9944
	Low NSES : rs2074351 (*PON1*)	12.35 (1.66 - 91.59)	0.0140
	rs2074351 (*PON1*) : High NSES	0.94 (0.13 - 6.94)	0.9515
	rs2074351 (*PON1*) : rs8073146 (*CRHR1*)	0.43 (0.04 - 4.20)	0.4643
	Low NSES : rs8073146 (*CRHR1*)	0.87 (0.07 - 10.79)	0.9157
	High NSES : rs8073146 (*CRHR1*)	0.35 (0.01 - 18.88)	0.6051
rs12621551 (*COL4A3*)	Low NSES	0.25 (0.05 - 1.35)	0.1070
	rs2074351 (*PON1*)	1.03 (0.32 - 3.33)	0.9572
	High NSES	0.49 (0.10 - 2.32)	0.3652
	rs12621551 (*COL4A3*)	0.65 (0.15 - 2.85)	0.5686
	Low NSES : rs2074351 (*PON1*)	10.03 (1.38 - 72.83)	0.0227
	rs2074351 (*PON1*) : High NSES	1.38 (0.21 - 9.30)	0.7399
	rs2074351 (*PON1*) : rs12621551 (*COL4A3*)	0.73 (0.14 - 3.81)	0.7077
	Low NSES : rs12621551 (*COL4A3*)	2.68 (0.35 - 20.54)	0.3414
	High NSES : rs12621551 (*COL4A3*)	1.48 (0.21 - 10.32)	0.6938
rs11680670 (*COL4A3*)	Low NSES	0.27 (0.04 - 1.63)	0.1525
	rs2074351 (*PON1*)	0.91 (0.29 - 2.90)	0.8780
	High NSES	0.41 (0.07 - 2.45)	0.3293
	rs11680670 (*COL4A3*)	0.67 (0.15 - 3.06)	0.6095
	Low NSES : rs2074351 (*PON1*)	10.68 (1.43 - 79.62)	0.0208
	rs2074351 (*PON1*) : High NSES	1.45 (0.20 - 10.36)	0.7134
	rs2074351 (*PON1*) : rs11680670 (*COL4A3*)	0.96 (0.19 - 5.02)	0.9654
	Low NSES : rs11680670 (*COL4A3*)	1.93 (0.27 - 13.97)	0.5132
	High NSES : rs11680670 (*COL4A3*)	2.00 (0.28 - 14.09)	0.4860

Specific covariate	Variable	Odds ratio (95% CI)	P Value
rs12621551 (*COL4A3*)	Low NSES	0.29 (0.06 - 1.44)	0.1294
	rs2074351 (*PON1*)	0.79 (0.26 - 2.34)	0.6672
	High NSES	0.38 (0.08 - 1.86)	0.2303
	rs12621551 (*COL4A3*)	1.03 (0.16 - 6.55)	0.9723
	Low NSES : rs2074351 (*PON1*)	10.59 (1.43 - 78.64)	0.0211
	rs2074351 (*PON1*) : High NSES	1.61 (0.23 - 11.52)	0.6326
	rs2074351 (*PON1*) : rs12621551 (*COL4A3*)	1.28 (0.16 - 9.97)	0.8156
	Low NSES : rs12621551 (*COL4A3*)	3.24 (0.19 - 54.20)	0.4142
	High NSES : rs12621551 (*COL4A3*)	3.41 (0.34 - 34.55)	0.2999
rs4845397 (*KCNN3*)	Low NSES	0.39 (0.08 - 1.91)	0.2452
	rs2074351 (*PON1*)	1.16 (0.37 - 3.66)	0.8048
	High NSES	0.53 (0.11 - 2.55)	0.4276
	rs4845397 (*KCNN3*)	1.73 (0.36 - 8.42)	0.4956
	Low NSES : rs2074351 (*PON1*)	13.18 (1.66 - 104.94)	0.0148
	rs2074351 (*PON1*) : High NSES	1.48 (0.22 - 9.98)	0.6846
	rs2074351 (*PON1*) : rs4845397 (*KCNN3*)	0.33 (0.05 - 2.05)	0.2340
	Low NSES : rs4845397 (*KCNN3*)	0.37 (0.04 - 3.83)	0.4055
	High NSES : rs4845397 (*KCNN3*)	0.89 (0.11 - 7.18)	0.9096
Urinary tract infection	Low NSES	0.08 (0.01 - 0.90)	0.0405
	rs2074351 (*PON1*)	1.30 (0.40 - 4.27)	0.6654
	High NSES	0.64 (0.13 - 3.01)	0.5680
	Urinary tract infection	1.69 (0.41 - 6.95)	0.4656
	Low NSES : rs2074351 (*PON1*)	22.48 (1.98 - 254.95)	0.0120
	rs2074351 (*PON1*) : High NSES	1.25 (0.18 - 8.56)	0.8180
	rs2074351 (*PON1*) : Urinary tract infection	0.27 (0.04 - 1.60)	0.1485

Specific covariate	Variable	Odds ratio (95% CI)	P Value
	Low NSES : Urinary tract infection	9.88 (0.99 - 98.84)	0.0513
	High NSES : Urinary tract infection	0.64 (0.06 - 6.57)	0.7081
Vaginal discharge	Low NSES	0.31 (0.06 - 1.62)	0.1630
	rs2074351 (*PON1*)	1.01 (0.32 - 3.20)	0.9907
	High NSES	0.37 (0.07 - 2.03)	0.2533
	Vaginal discharge	1.31 (0.32 - 5.29)	0.7047
	Low NSES : rs2074351 (*PON1*)	10.72 (1.46 - 78.66)	0.0197
	rs2074351 (*PON1*) : High NSES	1.58 (0.23 - 10.87)	0.6397
	rs2074351 (*PON1*) : Vaginal discharge	0.57 (0.11 - 2.89)	0.4937
	Low NSES : Vaginal discharge	1.45 (0.19 - 10.82)	0.7174
	High NSES : Vaginal discharge	2.16 (0.31 - 14.94)	0.4359
